# Clinical utility of CSF metagenomic next-generation sequencing in suspected CNS infection: performance against a composite reference standard and read-count stratification

**DOI:** 10.3389/fcimb.2026.1905481

**Published:** 2026-07-28

**Authors:** Peng Shi, Zhongzheng Liu, Xinyu Wu, FangYu Zhao, Juanjuan Xu, Qianqian Li, Ming Ye, Di Nian

**Affiliations:** 1 Department of Neurology, The First Affiliated Hospital of Bengbu Medical University, Bengbu, China; 2 Department of Clinical Laboratory, Anhui Province Key Laboratory of Basic and Translational Research of Inflammation-related Diseases, First Affiliated Hospital of Bengbu Medical University, Bengbu, Anhui, China; 3 Department of Medical Examination, Bengbu Medical University, Bengbu, China

**Keywords:** central nervous system infection, cerebrospinal fluid, diagnostic performance, interpretation framework, metagenomic next-generation sequencing, read count

## Abstract

**Background:**

Metagenomic next-generation sequencing (mNGS) of cerebrospinal fluid (CSF) is increasingly used to identify pathogens in suspected central nervous system (CNS) infections. However, integrating these results into real-world clinical decision-making remains problematic, particularly given the lack of standardized quantitative metrics beyond raw read counts.

**Methods:**

We retrospectively analyzed 46 patients with suspected encephalitis, meningitis, or meningoencephalitis who underwent CSF mNGS testing. Etiologic certainty was classified using a composite clinical reference standard as Definite, Probable, or Unlikely. We assessed concordance between mNGS findings and the Likely etiology category (Definite or Probable), calculated diagnostic performance metrics, characterized the detected pathogens, and explored a tiered interpretation framework based on maximum read counts per patient (<10, 10–49, and >=50). Trends across read-count strata were evaluated using the Cochran-Armitage test, and exact binomial 95% confidence intervals (CIs) were calculated.

**Results:**

CSF mNGS detected pathogens in 13 of 46 patients (28.3%). Positivity increased with greater adjudicated diagnostic certainty, from 0% in Unlikely cases to 11.8% in Probable cases and 73.3% in Definite cases. Within the composite reference framework, mNGS showed 40.6% sensitivity, 100% specificity, 100% positive predictive value, and 42.4% negative predictive value, indicating stronger rule-in than rule-out performance. Viral detections predominated, with herpes simplex virus type 1 and varicella-zoster virus as the most frequent pathogens; all findings should be interpreted in the context of DNA-only testing. Among mNGS-positive patients with Likely etiologies, the proportion classified as Definite increased across higher max-read strata, but these tier-specific estimates were imprecise and should be viewed as exploratory.

**Conclusion:**

In this real-world cohort, positive CSF mNGS results supported an infectious etiology more strongly than negative results excluded it. Max-read-based stratification may have exploratory interpretive value for positive findings, but it should not be considered a validated clinical decision rule and requires confirmation in larger multicenter studies with standardized reference standards.

## 1 Introduction

Central nervous system (CNS) infections, whether presenting as encephalitis, meningitis, or meningoencephalitis demand rapid therapeutic decisions. The window for initiating specific antiviral or antibacterial therapy is narrow, yet clinical syndromes often lack specificity, overlapping with autoimmune encephalitides, malignancies, and other inflammatory conditions (Volpe, 2022). This diagnostic ambiguity forces clinicians to commit to empiric broad-spectrum treatment before microbiologic confirmation becomes available, contributing to unnecessary antimicrobial exposure and potentially delaying appropriate care (Ramachandran and Wilson, 2020).

Traditional CSF diagnostics remain constrained by low pathogen biomass, limited specimen availability, fastidious growth requirements, and the ubiquitous reality of pre-sampling antibiotics. Even targeted PCR assays require foreknowledge of suspected pathogens and inevitably miss unexpected or novel etiologies. These persistent limitations have fueled interest in unbiased detection strategies capable of complementing—rather than replacing—conventional testing. Indeed, clinical metagenomic sequencing has shown promise in prospective cohorts of suspected meningitis and encephalitis, identifying diverse pathogens missed by standard panels in a single unified assay (Wilson et al., 2019).

mNGS has accordingly gained traction as an adjunctive tool for CNS infection diagnosis. Multicenter experience suggests its utility varies by pathogen class and quantitative signal intensity (Xing et al., 2020). However, reported detection rates differ substantially across institutions, reflecting heterogeneity in patient selection, laboratory protocols, and case definitions (Shean et al., 2024). Notably, technical variables—such as whether workflows target cell-free versus cellular DNA—can markedly influence sensitivity (Yu et al., 2022), underscoring that mNGS encompasses diverse methodological implementations rather than a standardized test.

More problematic than technical variability is the challenge of clinical interpretation (Chen et al., 2024; Wu et al., 2024). Binary positive or negative reporting proves insufficient in practice: low-abundance sequences may represent contamination or colonization; polymicrobial detections complicate attribution; and negative results risk providing false reassurance when clinical suspicion remains high. Analyses of discordance between mNGS results and final clinical diagnoses reveal frequent inconsistencies and a critical absence of standardized frameworks for adjudicating false positives or integrating findings into bedside decision-making (Wang et al., 2022). These interpretive difficulties are compounded when laboratories report only raw read counts without standardized background controls or relative abundance metrics.

In this study, we evaluated CSF mNGS in a consecutive real-world cohort of adults with suspected CNS infection. Our objectives were to describe pathogen detection patterns, estimate diagnostic performance against a composite clinical reference standard, and explore whether a simple patient-level max-read stratification might provide a cautious interpretive framework for positive results. Because the reference process was not independent of routine-care mNGS findings and the number of positive cases was limited, all threshold-based inferences were treated as exploratory rather than confirmatory.

## 2 Materials and methods

### 2.1 Study design

We retrospectively identified consecutive adults who underwent CSF metagenomic next-generation sequencing at the First Affiliated Hospital of Bengbu Medical University between May 2024 and June 2025 as part of the clinical evaluation of suspected CNS infection. Medical records and sequencing reports were retrieved from the hospital information system and the mNGS reporting platform, respectively. The investigation followed the Declaration of Helsinki guidelines, all data were anonymized prior to analysis, and no research procedure interfered with clinical decision-making. The institutional ethics committee of the First Affiliated Hospital of Bengbu Medical University approved the protocol (approved number: [2023]KY036).

### 2.2 Inclusion and exclusion criteria

Eligible participants were adults aged 18–80 years admitted with suspected acute CNS infection (encephalitis, meningitis, or meningoencephalitis). The treating team had documented an acute neurological infectious syndrome-characterized by fever, headache or meningismus, altered mental status, focal deficits, or new-onset seizures-supported by compatible CSF and/or neuroimaging findings when available. Inclusion also required>=2 mL of residual CSF for metagenomic sequencing and sufficient clinical documentation to permit etiologic adjudication.

We excluded individuals with contraindications to lumbar puncture, specifically coagulopathy (international normalized ratio>1.5 or platelet count<50×10^9^/L) or radiologic evidence of impending brain herniation. Other exclusion criteria comprised immunocompromise (HIV infection, solid-organ transplantation, systemic chemotherapy within 6 months, or prolonged high-dose corticosteroids>0.5 mg/kg/day prednisone equivalent for>30 days); established alternative non-infectious diagnoses (autoimmune encephalitis, acute ischemic stroke, intracranial neoplasm, or metabolic encephalopathy); pregnancy or lactation; and severe comorbidities predicting survival<3 months.

### 2.3 Clinical data collection and definitions

From the hospital’s medical record system, we abstracted demographic characteristics, comorbid conditions, presenting manifestations, and initial laboratory values. Neuroimaging reports were reviewed when performed. Hematologic assessment comprised complete blood count with differential (absolute and relative counts of granulocytes, lymphocytes, monocytes, and eosinophils) along with standard inflammatory markers: C-reactive protein, procalcitonin, and D-dimer. Histopathological findings, when obtained, were also extracted.

Reflecting routine clinical practice in this cohort, clinicians generally withheld systemic antimicrobials pending initial blood cultures, yet empiric therapy was frequently started before lumbar puncture and CSF acquisition for sequencing. We categorized patients according to whether they received any systemic antibacterial, antiviral, or antifungal agent prior to CSF sampling for mNGS (binary yes or no). However, the retrospective record did not provide uniformly granular information on drug class, duration, or timing for all patients, limiting detailed subgroup analysis.

### 2.4 CSF sampling and conventional microbiological tests

Physicians obtained cerebrospinal fluid via lumbar puncture under local anesthesia (2% lidocaine) using 20–22G spinal needles. Approximately 4 mL was collected aseptically and allocated between metagenomic sequencing and standard laboratory analyses (cell count and cytology). We took precautions to minimize blood contamination during the procedure.

Automated cell counts were performed on the Sysmex XN-3100 analyzer (Sysmex, America), with manual differentials examined under high-power microscopy (×40). White blood cell concentration was calculated as: WBC/μL= (total cells in four large squares/4) ×dilution factor×10, reported as cells/μL (or×10^6^/L).

According to clinical indications, routine microbiological examinations were arranged, including blood culture and CSF antigen testing. Targeted CSF PCR is not common in our environment, so it cannot be used as a systematic reference method.

### 2.5 mNGS workflow and reporting metrics

We shipped CSF specimens on ice and processed them within an hour of collection. Microbial DNA was extracted and host background depleted using the FastPure Host Removal and Microbiome DNA Isolation Kit (DC501, Vazyme, China). Following quantification on the Qubit 4.0 fluorometer (Q33226, Thermo-Fisher, America), we prepared sequencing libraries with UltraClean Universal Plus DNA Library Prep Kit for MGI (UNDM637, Vazyme, China), subjected them to quality assessment, denatured the products to single strands, and amplified them by rolling-circle replication to generate DNA nanoballs.

Sequencing employed the DNBSEQ-200 platform (MGI, China) with combinatorial probe-anchor synthesis (cPAS) chemistry, generating paired-end 50-bp reads. The bioinformatics pipeline discarded low-quality and adapter-contaminated sequences, stripped human reads, and aligned the remaining clean data to a curated commercial database spanning 24,142 taxa: 11,836 bacteria, 11,021 viruses, 1,872 fungi, 421 parasites, 153 mycobacteria, 118 Mycoplasma/Chlamydia, and 105 Rickettsia. We excluded likely background organisms—including laboratory contaminants, environmental species, and common commensals—according to the laboratory’s validated filtering protocol before final reporting.

For this analysis, we classified an mNGS report as “positive” when the pipeline identified any microorganism after QC and background filtering, irrespective of read count. Reports yielding no microbial hits were classified as “negative”.

### 2.6 Quantitative metrics and handling of microbial results

Our primary quantitative measure was the maximum read count per patient (hereafter, max reads), representing the highest read depth observed for any single organism within an individual’s CSF mNGS profile. When a patient’s report contained multiple microbial hits, we assigned the read count of the most abundant species as the max reads value for that individual.

To characterize the pathogen landscape, we tabulated detection events (each individual microbial identification) and, where relevant, aggregated findings at the patient level (counting each participant only once per organism).

### 2.7 Etiology adjudication

We convened an expert panel comprising specialists in infectious diseases, neurology, and clinical microbiology to determine the final diagnosis for each case. Panelists independently reviewed all available clinical records, laboratory values, neuroimaging, and microbiological findings, with a final category assigned when at least two of the three experts reached consensus.

We classified cases into three levels of diagnostic certainty:

Definite etiology: Strong evidence supported a specific infectious agent, including definitive microbiological or pathological confirmation (e.g., positive culture or antigen detection), or marked clinical concordance with a particular pathogen accompanied by compatible disease trajectory and treatment response.

Probable etiology: The clinical presentation, imaging findings, and CSF profile strongly suggested CNS infection, though definitive pathogen identification remained elusive.

Unlikely etiology: Clinical evidence did not support an infectious process; alternative non-infectious diagnoses appeared more probable, or follow-up data contradicted the diagnosis of CNS infection.

For analytical purposes, Definite and Probable categories were combined as Likely etiology. Because the study was retrospective and mNGS formed part of routine care, adjudicators were not blinded to sequencing results. Accordingly, the composite reference standard was not fully independent of the index test, and incorporation bias may have inflated observed concordance and apparent specificity.

### 2.8 Read-count stratification

Among mNGS-positive patients, we grouped max reads into three pragmatic categories: <10, 10-49, and >=50. These thresholds were selected for descriptive interpretive purposes because raw read counts are routinely reported and easy to recognize clinically. They were not intended as validated or universally generalizable clinical decision cutoffs. Within each layer, we calculated the definite-etiology probability as the proportion of adjudicated Definite cases divided by the total number of cases in that stratum, reporting exact 95% confidence intervals (CIs).

### 2.9 Outcome measures

Primary outcomes were the overall positivity rate of CSF mNGS and the apparent diagnostic performance of mNGS against the composite Likely etiology reference, expressed as sensitivity, specificity, positive predictive value, and negative predictive value with exact 95% confidence intervals. Secondary analyses described positivity across adjudication categories, summarized the pathogen spectrum, and explored the proportion of Definite etiologies within each read-count stratum. Because cell counts were small and the stratified analyses were exploratory, exact methods were used for interval estimation, no complex modeling was performed, and no multiple-comparison adjustment was applied.

### 2.10 Statistical analysis

We expressed categorical variables as frequencies and percentages. For continuous variables, we reported either medians with interquartile ranges or means with standard deviations, depending on distributional properties.

For diagnostic accuracy, we cross-tabulated mNGS results against the composite Likely etiology reference (Definite or Probable) to generate a 2×2 table. From this, we derived sensitivity, specificity, and predictive values, each accompanied by 95% confidence intervals. Because cell frequencies were small and some strata contained zero events (for example, no mNGS detections in the Unlikely group), we computed exact binomial confidence limits (Clopper–Pearson) rather than asymptotic approximations.

Among mNGS-positive cases, we estimated the proportion of Definite etiologies within each max-read stratum, using exact binomial 95% confidence intervals. We tested for a linear trend in these proportions across ascending max-read categories with the Cochran–Armitage statistic (Neuhäuser and Hothorn, 1999). We regarded two-sided p values <0.05 as statistically significant. All analyses were conducted in R version 4.4.2 (Team, Core, 2012).

## 3 Results

### 3.1 Baseline characteristics and overall mNGS results

We recruited 46 patients who underwent CSF metagenomic sequencing, of whom 33 yielded negative results (71.7%) and 13 positive results (28.3%). The cohort had a median age of 47 years (IQR 32–65), included 18 women (39%), and comprised 41 first-visit cases (89%). All specimens underwent DNA-based testing (**Table 1**).

**Table 1:** Baseline clinical characteristics and laboratory findings.

Characteristic	Overall N = 46^1^	Negative N = 33^1^	Positive N = 13^1^	P-value^1^
age	47 (32, 65)	44 (28, 65)	50 (35, 58)	0.6
gender				>0.9
Female	18 (39%)	13 (39%)	5 (38%)	
male	28 (61%)	20 (61%)	8 (62%)	
first_visit	41 (89%)	29 (88%)	12 (92%)	>0.9
dna_rna				
DNA	46 (100%)	33 (100%)	13 (100%)	
csf_wbc	2 (2, 10)	2 (2, 8)	2 (2, 39)	0.6
wbc	7.8 (6.8, 9.5)	7.5 (6.4, 9.2)	8.8 (7.0, 10.6)	0.2
neu_abs	5.6 (4.1, 7.6)	5.3 (4.1, 7.4)	6.4 (4.6, 8.5)	0.5
lym_abs	1.59 (1.19, 1.96)	1.36 (1.10, 1.87)	1.77 (1.63, 2.25)	0.028
mono_abs	0.50 (0.37, 0.71)	0.45 (0.34, 0.72)	0.52 (0.50, 0.66)	0.4
crp	6 (5, 12)	6 (5, 11)	7 (5, 17)	0.5
Unknown	1	0	1	
pct_ml_nal				>0.9
5	1 (17%)	0 (0%)	1 (25%)	
7	1 (17%)	1 (50%)	0 (0%)	
18	1 (17%)	0 (0%)	1 (25%)	
20	1 (17%)	0 (0%)	1 (25%)	
40	1 (17%)	0 (0%)	1 (25%)	
89	1 (17%)	1 (50%)	0 (0%)	
Unknown	40	31	9	
hb	132 (124, 148)	131 (124, 148)	132 (129, 149)	0.7
rbc	4.41 (4.10, 4.92)	4.41 (4.10, 4.81)	4.48 (4.38, 5.06)	0.4

^1^ Median (Q1, Q3); n (%); ^2^Wilcoxon rank sum test; Fisher’s exact test; NA; Wilcoxon rank sum exact test.

The mNGS-positive and negative groups showed similar demographic characteristics. There were no inter group differences in age, gender, and first visit status (median age 50 years [IQR 35-58] vs 44 years [28-65], P = 0.6; 38% for females compared to 39%, P>0.9; 92% of first-time patients compared to 88%, P>0.05). The cerebrospinal fluid white blood cell count is also comparable (median 2×10^6^/L [IQR 2-39] and 2×10^6^/L [2-8], P = 0.6) (**Table 1**).

Peripheral white blood cell and absolute neutrophil counts trended higher in the mNGS-positive arm, though neither reached statistical significance (WBC 8.8×10^9^/L, IQR 7.0–10.6 vs 7.5×10^9^/L, 6.4-9.2, p=0.2; neutrophils 6.4×10^9^/L, 4.6-8.5, vs 5.3×10^9^/L, 4.1-7.4, p=0.5). However, absolute lymphocyte counts were significantly elevated in the positive group (1.77×10^9^/L, IQR 1.63-2.25, vs 1.36×10^9^/L, 1.10-1.87, p=0.028). Monocyte counts, C-reactive protein, hemoglobin, and erythrocyte counts did not differ between groups (all p>0.05). Pre-sampling anti-infective exposure was common in routine care, although the retrospective documentation was not sufficiently detailed to support stable drug-class-specific subgroup comparisons.

### 3.2 Etiology adjudication and concordance between mNGS and likely etiology

Among the 46 patients, etiology adjudication classified 14 (30.4%) as Unlikely etiology, 17 (37.0%) as Probable etiology, and 15 (32.6%) as Definite etiology (**Table 2**). Probable and Definite categories were combined as “Likely etiology” (n=32, 69.6%), whereas Unlikely etiology constituted the unlikely group (n=14, 30.4%).

**Table 2:** Etiology distribution.

Etiology_class	n	Percent
Unlikely Etiology	14	30.40%
Probable Etiology	17	37.00%
Definite Etiology	15	32.60%

**Figure 1:**
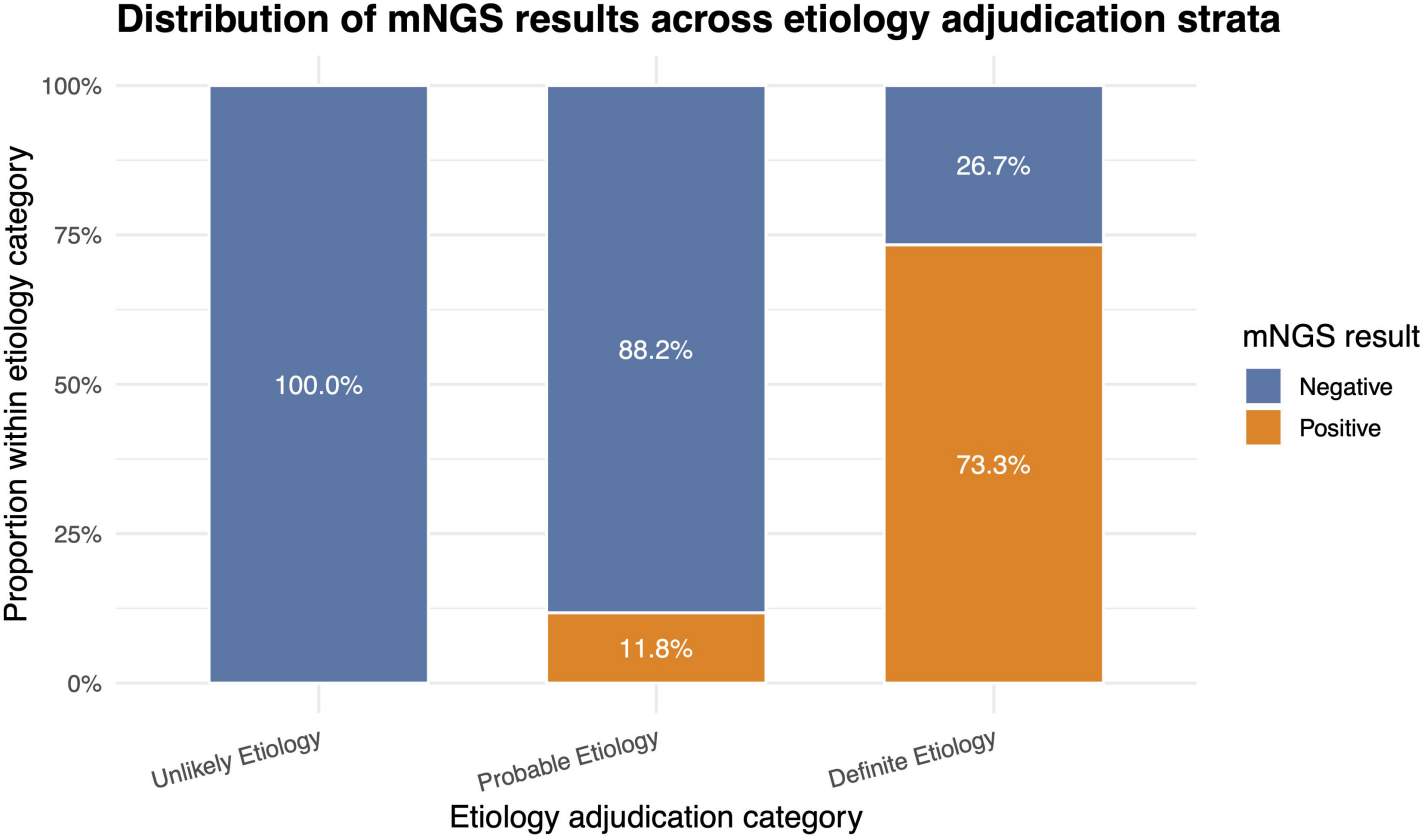
Distribution of CSF mNGS results across etiology adjudication strata. A 100% stacked bar plot shows the proportion of mNGS-positive and mNGS-negative patients within each adjudicated category (Unlikely, Probable, and Definite) determined by the composite clinical reference standard. Percentages are calculated within each etiology stratum (row percentages). Numbers on or above bars indicate the corresponding patient counts.

A marked distribution shift was observed across etiology strata: mNGS positivity was predominantly enriched in the Definite etiology group (mNGS positive in 73.3% of Definite cases), whereas the mNGS positivity rate was 11.8% in the Probable group and 0% in the Unlikely group (**Figure 1**). Overall, the mNGS positivity rate was 28.3% (13/46), and all mNGS-positive cases were adjudicated as likely etiology (13/13, 100%) (**Table 3**).

**Table 3:** Concordance between CSF mNGS results and etiology adjudication using a composite clinical reference standard.

Reference Standard	Test + (mNGS positive)	Test - (mNGS negative)	Metric	Estimate	95% confidence
Disease + (Likely)	13	19	Sensitivity	40.6%	23.7%–59.4%
Disease - (Unlikely)	0	14	Specificity	100%	76.8%–100%
			PPV	100%	75.3%–100%
			NPV	42.4%	25.5%–60.8%

Left table shows the 2×2 contingency table with “Likely” (Definite/Probable) as the condition-positive reference and “Unlikely” as condition-negative. Right table reports diagnostic performance metrics (sensitivity, specificity, positive predictive value PPV, and negative predictive value NPV) with 95% confidence intervals (CIs). Confidence intervals were calculated using exact (Clopper–Pearson) methods where applicable due to small sample size and the presence of zero cells.

Using likely etiology as the reference standard (disease positive) and mNGS positivity as test positive, the sensitivity of mNGS for identifying likely etiology was 40.6% (13/32; 95% CI, 23.7%–59.4%), and specificity was 100% (14/14; 95% CI, 76.8%–100%). The positive predictive value (PPV) was 100% (13/13; 95% CI, 75.3%–100%), and the negative predictive value (NPV) was 42.4% (14/33; 95% CI, 25.5%–60.8%) (**Table 3**). Notably, 19 (57.6%) of mNGS-negative cases were still adjudicated as likely etiology, indicating that a negative mNGS result did not reliably exclude a likely infectious etiology in this cohort (**Table 3**). Given the limited sample size and the absence of false-positive results (FP = 0), point estimates for specificity and PPV were 100%; however, the corresponding confidence intervals remained wide, warranting validation in larger cohorts.

### 3.3 Pathogen landscape and quantitative signals in positive cases

**Figure 2:**
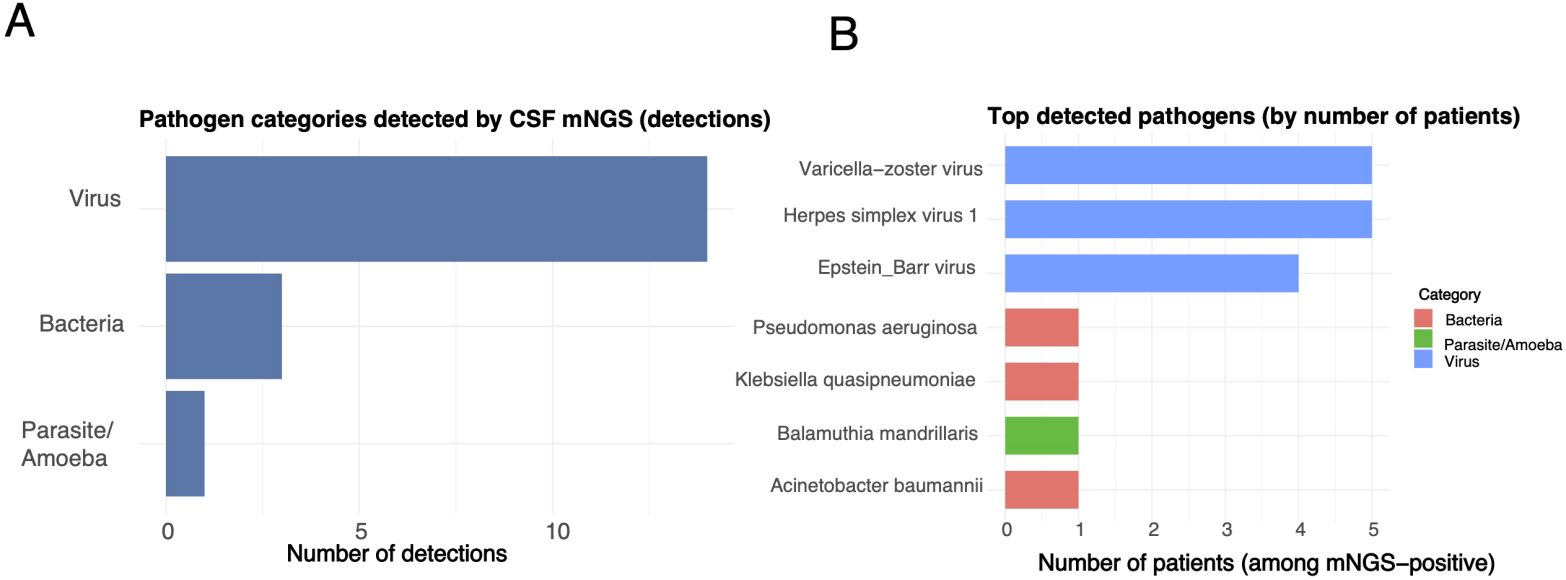
Pathogen spectrum among mNGS-positive patients. **(A)** Distribution of detected organisms by pathogen group (virus, bacteria, fungi, parasite) summarized as detection events. **(B)** Most frequently detected organisms summarized at the patient level. Because multiple organisms could be reported for a single patient, totals in detection-event analyses may exceed the number of patients.

Thirteen patients yielded positive sequencing results, collectively generating 18 microbial detections. Viruses predominated, constituting 77.8% (14/18) of all hits, followed by bacteria at 16.7% (3/18) and protozoa at 5.6% (1/18) (**Figure 2**). Herpes simplex virus 1, Varicella-zoster virus, and Epstein–Barr virus were the most prevalent agents, each identified in multiple individuals (**Table 4**). Specifically, HSV-1 and VZV were each detected in 5 patients, while EBV appeared in 4. Bacterial isolates comprised *Acinetobacter baumannii*, *Pseudomonas aeruginosa*, and *Klebsiella quasipneumoniae* (one case each). A single amoebic pathogen, *Balamuthia mandrillaris*, was also identified.

**Table 4:** Detected organisms and read counts in mNGS-positive patients.

Pathogen	Category	Patients, n (%)	Detections, n	Reads, median (IQR)
Herpes simplex virus 1	Virus	5 (38.5%)	5	35.0 (2.0-58.0)
Varicella-zoster virus	Virus	5 (38.5%)	5	16.0 (1.0-26.0)
Epstein_Barr virus	Virus	4 (30.8%)	4	22.5 (3.0-50.2)
Klebsiella quasipneumoniae	Bacteria	1 (7.7%)	1	236.0 (236.0-236.0)
Balamuthia mandrillaris	Parasite/Amoeba	1 (7.7%)	1	216.0 (216.0-216.0)
Acinetobacter baumannii	Bacteria	1 (7.7%)	1	73.0 (73.0-73.0)
Pseudomonas aeruginosa	Bacteria	1 (7.7%)	1	10.0 (10.0-10.0)

Most infected individuals carried a single pathogen (*n* = 9, 69.2%), while four (30.8%) showed polymicrobial infection. The median microbial burden per positive sample was one species (IQR 1–2), ranging from one to three (**Table 5**).

**Table 5:** Distribution of detected pathogens by mNGS (single vs. multiple pathogens).

Pathogens_group	Number	Percent
Multiple pathogens	4	0.308
Single pathogen	9	0.692

median_n(1); q1(1); q3(2); min_n(1); max_n(3).

**Figure 3:**
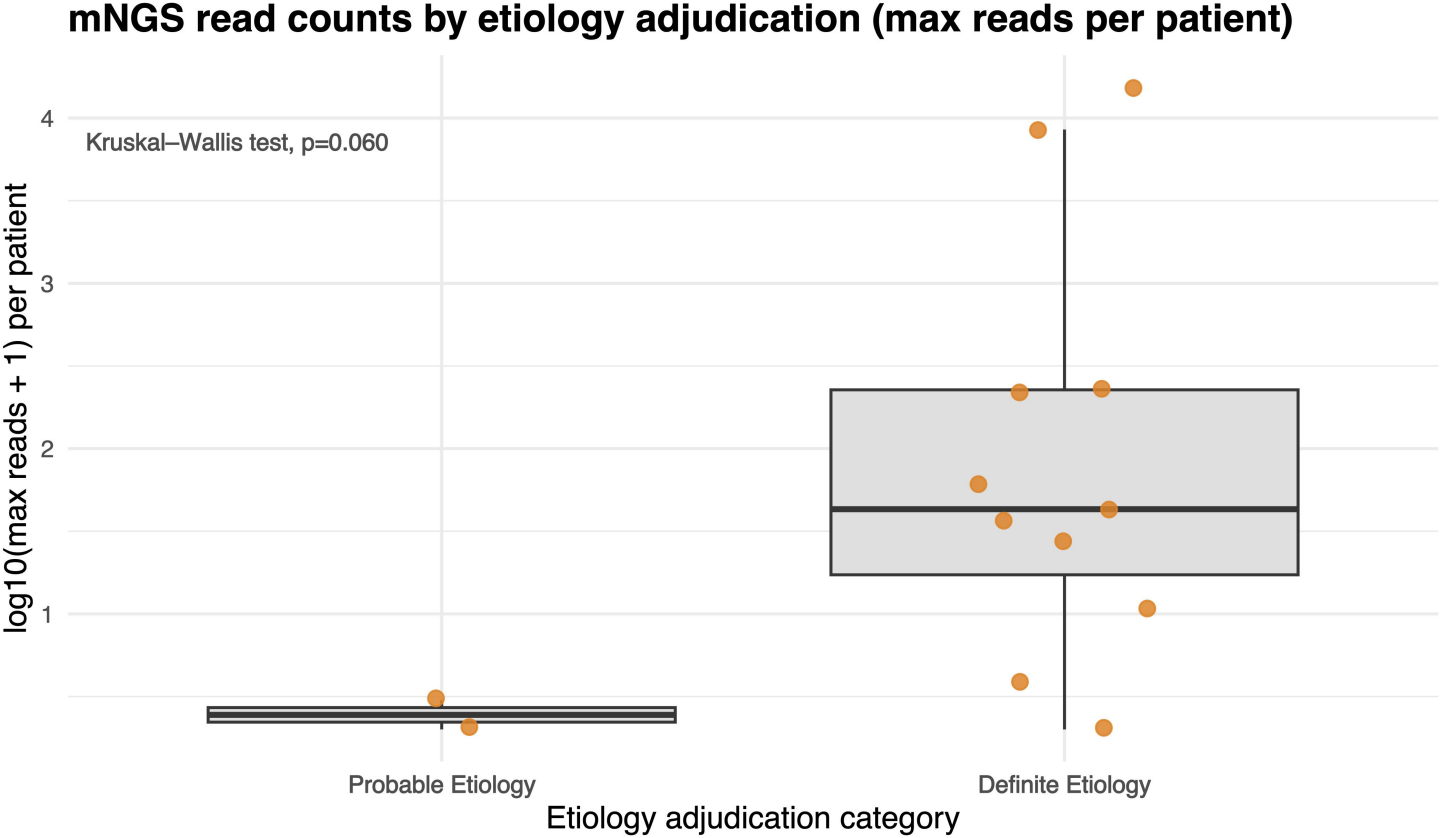
Distribution of max reads per patient across etiology adjudication categories. Each point represents one patient’s max reads per patient (defined as the highest read count among all reported organisms in that patient’s CSF mNGS report). The y-axis is shown as log10(max reads+1). Boxplots indicate median and interquartile range (IQR), with whiskers representing 1.5×IQR. Differences across categories (Definite, Probable) were assessed using the Kruskal–Wallis test.

We next examined whether read-count magnitude tracked with diagnostic certainty. Stratifying the 13 positive cases by adjudicated category (Definite versus Probable), we found that maximum read counts trended higher in the Definite group (Kruskal–Wallis *P* = 0.060), approaching statistical significance (**Figure 3**). This pattern raises the possibility that elevated quantitative signals align with confirmed infection.

### 3.4 Association between fixed-threshold read-count strata and etiology certainty

**Figure 4:**
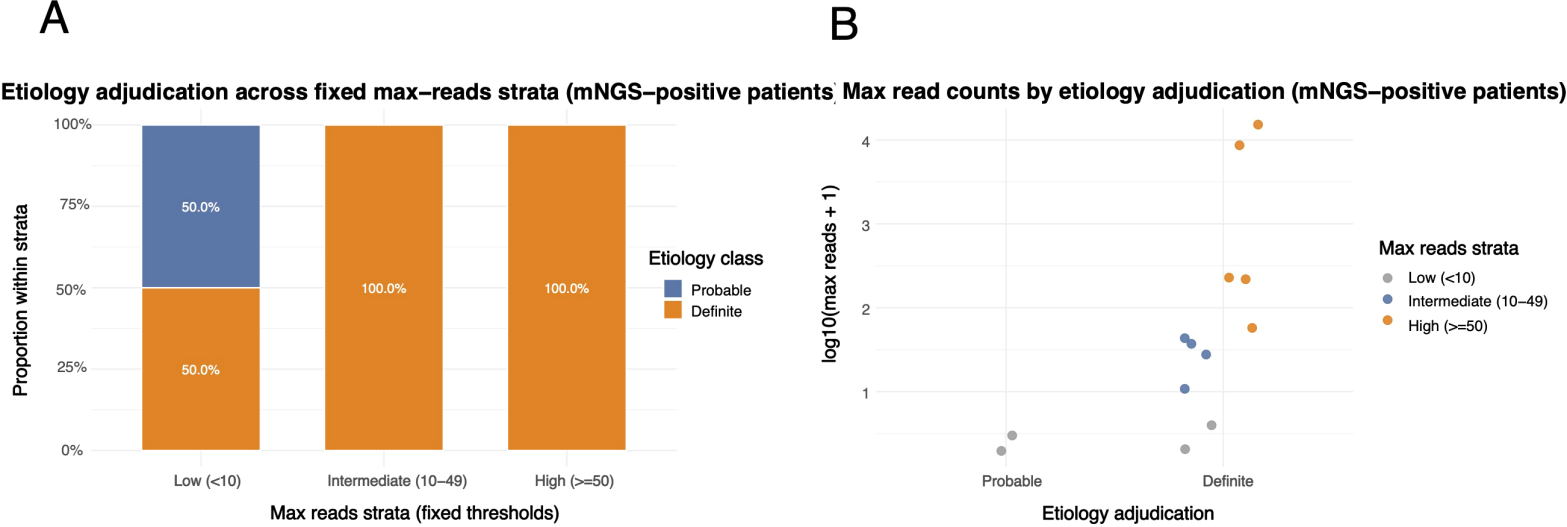
Max reads by etiology adjudication. **(A)** Etiology adjudication across fixed max reads per patient strata among mNGS-positive patients. A stacked bar plot shows the proportion of Definite and Probable etiology within each read-count stratum (<10, 10–49, and >=50). Percentages are calculated within each stratum. Trend in the proportion of Definite etiology across ordered strata was assessed using the Cochran-Armitage trend test. **(B)** Patient-level distribution of max reads per patient by etiology adjudication category. Each point represents one mNGS-positive patient, plotted by etiology category (Definite vs Probable) and log10(max reads+1) on the y-axis. Points are colored according to the pre-specified max-read strata (<10, 10-49, and>=50) to illustrate individual-level dispersion within and across categories.

To examine how quantitative signals relate to diagnostic confidence, we stratified the 13 positive cases by maximum read count into three pragmatic tiers: low (<10), moderate (10–49), and high (≥50) (**Table 6**). The proportion of Definite versus Probable diagnoses varied systematically with these quantitative levels. In the low tier (*n* = 4), only half the cases were judged Definite (2/4, 50%). By contrast, every case in the moderate (*n* = 4) and high (*n* = 5) tiers received a Definite classification (100% each) (**Table 6**), yielding a statistically significant stepwise trend (Cochran–Armitage test, *P* = 0.046) (**Figure 4**).

**Table 6:** Fixed-threshold max-read strata and etiology adjudication among mNGS-positive patients.

Max reads stratum	Probable, n (%)	Definite, n (%)	Total, n
Low (<10)	2 (50.0%)	2 (50.0%)	4
Intermediate (10–49)	NA	4 (100.0%)	4
High (≥50)	NA	5 (100.0%)	5

max reads per patient was defined as the highest read count among all reported organisms in a patient’s CSF mNGS report.

These data imply that when platforms report raw read counts, signal magnitude can gauge the reliability of a positive call. Intermediate and high read counts aligned consistently with confirmed infection, whereas low-count positives appeared less certain and benefit from corroborating clinical data.

### 3.5 Quantitative validation of a read-count–stratified interpretation framework

**Figure 5:**
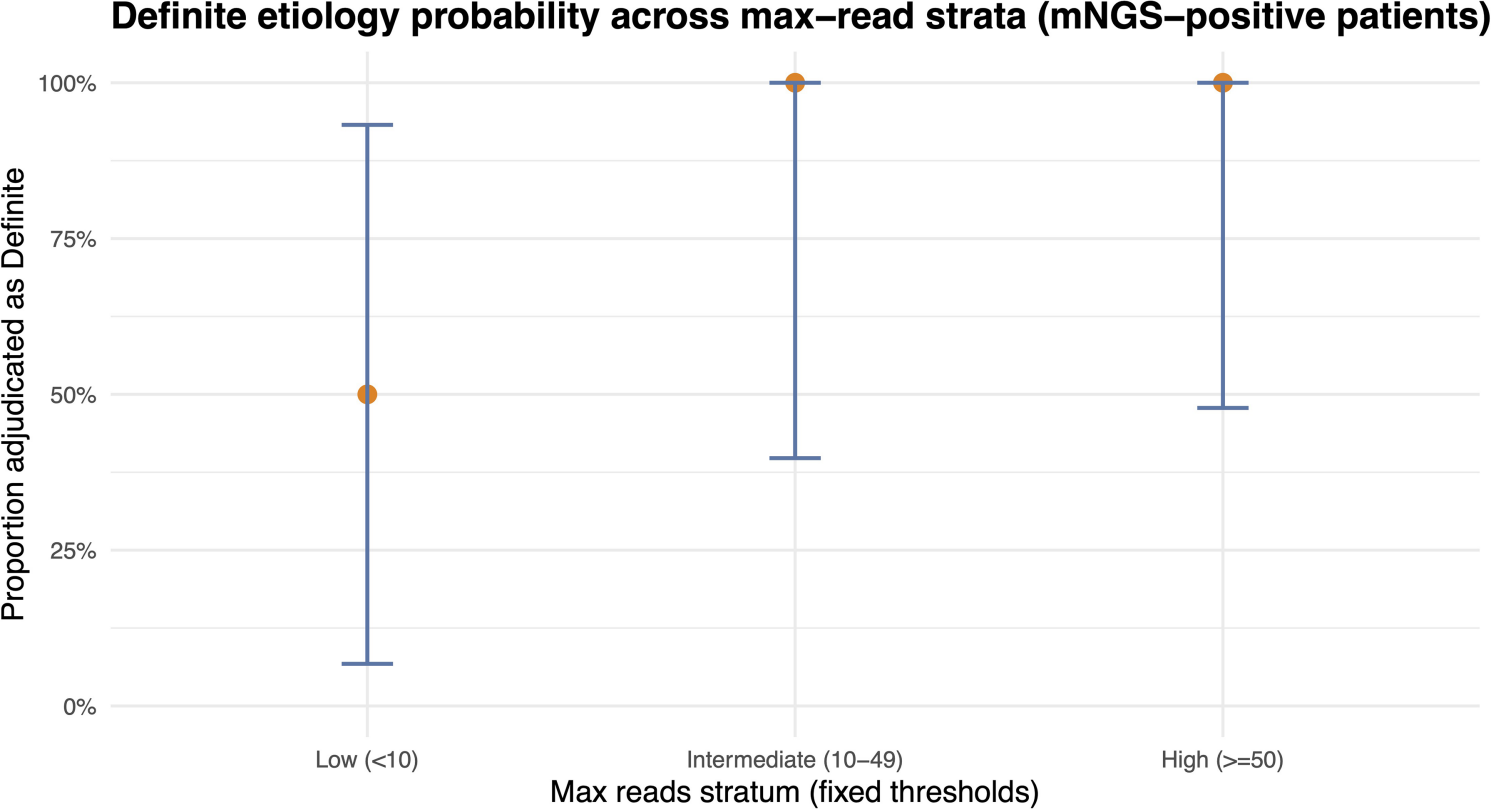
Probability of Definite etiology across max reads per patient strata among mNGS-positive patients. Points indicate the estimated proportion of Definite etiology within each stratum (<10, 10–49, and ≥50), and error bars indicate exact 95% confidence intervals (CIs) calculated using the binomial distribution.

To improve the clinical interpretability of positive mNGS results, we stratified mNGS-positive cases using fixed thresholds based on the maximum read count per patient (max reads): low (<10), intermediate (10-49), and high (>=50), and quantified the probability of Definite etiology within each tier (**Table 7**). In the low-read tier (n=4), the proportion adjudicated as Definite etiology was 50.0% (2/4; 95% CI, 6.8%–93.2%). In contrast, Definite etiology occurred in 100% of cases in both the intermediate and high tiers (4/4; 95% CI, 39.8%–100.0% and 5/5; 95% CI, 47.8%–100.0%, respectively) (**Table 7**). Collectively, these findings suggest that, in this cohort, mNGS-positive results with max reads ≥10 were highly concordant with higher-certainty etiology adjudication, whereas low-read positives (max reads <10) were less definitive and may warrant more cautious interpretation in conjunction with clinical evidence (**Figure 5**).

**Table 7:** Probability of Definite etiology across max-read strata with exact 95% confidence intervals.

Max reads stratum	Patients, n	Definite, n (%)	Probable, n (%)	Definite probability, % (95% CI)	Coverage among mNGS-positive	Coverage in total cohort
Low (<10)	4	2 (50.0%)	2 (50.0%)	50.0% (6.8–93.2)	4/13 (30.8%)	4/46 (8.7%)
Intermediate (10–49)	4	4 (100.0%)	0 (0.0%)	100.0% (39.8–100.0)	4/13 (30.8%)	4/46 (8.7%)
High (≥50)	5	5 (100.0%)	0 (0.0%)	100.0% (47.8–100.0)	5/13 (38.5%)	5/46 (10.9%)

For each stratum, we report the total number of patients, counts and proportions of Definite and Probable etiology, and the estimated probability of Definite etiology with exact 95% confidence intervals (CIs) calculated using the binomial distribution. Where applicable, additional summary measures (e.g., proportion of all mNGS-positive patients or of the total cohort) are provided to facilitate interpretation.

**Figure 6:**
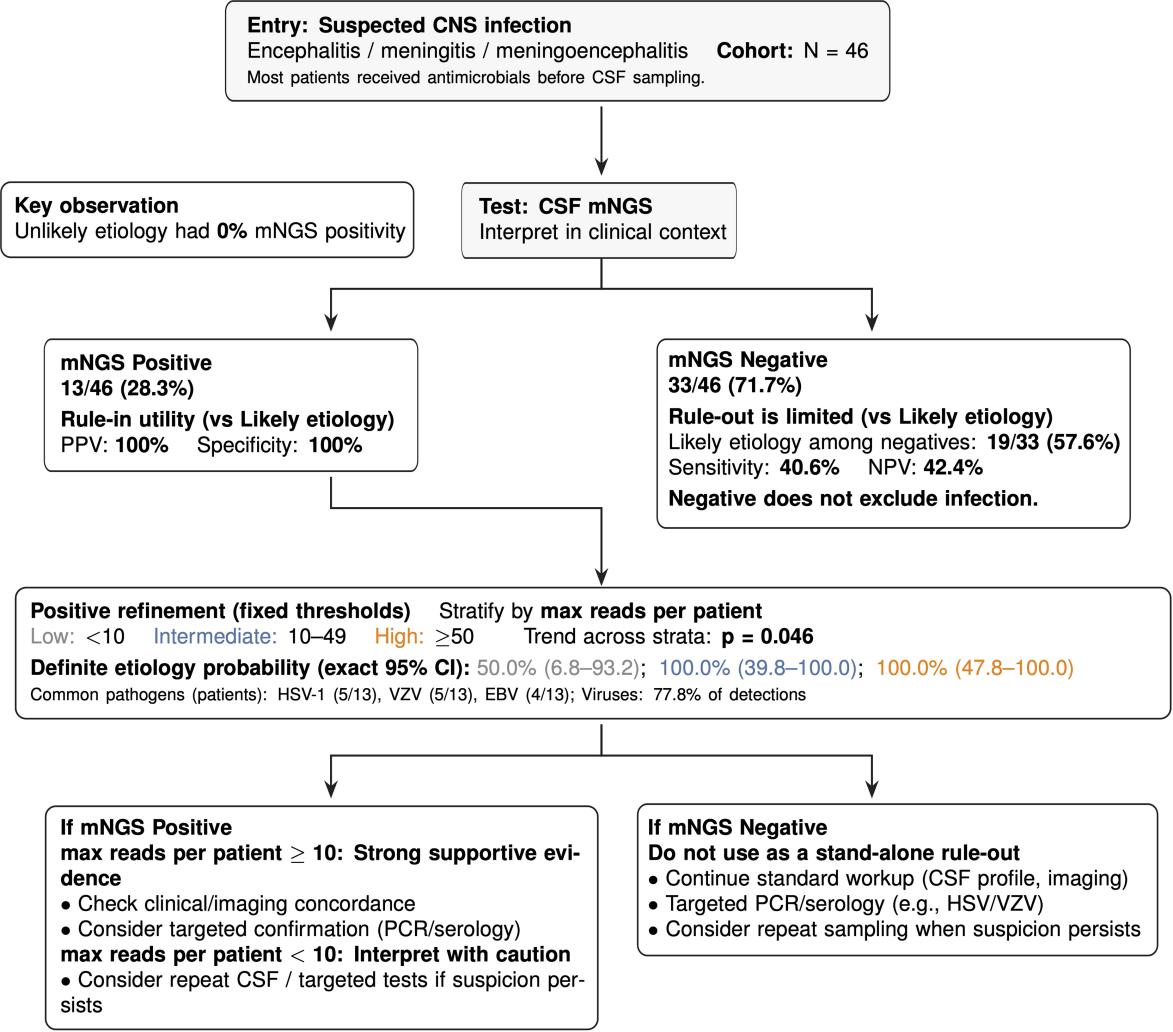
Proposed read-count–stratified interpretation pathway for CSF mNGS in suspected CNS infection. The workflow summarizes a pragmatic approach to integrating CSF mNGS with clinical adjudication in routine practice. Negative mNGS results should not be used as a stand-alone rule-out for infection and warrant continued standard workup and targeted testing when clinical suspicion persists. For mNGS positive cases, results are further graded using fixed thresholds of max reads per patient (<10, 10–49, and ≥50) to reflect the observed read-count–dependent concordance with higher-certainty etiology adjudication. “Max reads per patient” was defined as the highest read count among all organisms reported in a patient’s CSF mNGS report.

To facilitate bedside translation of these quantitative findings, we proposed a pragmatic interpretation pathway that integrates mNGS positivity or negativity with fixed max-read strata to grade the strength of supportive evidence and to guide confirmatory testing when needed (**Figure 6**). This workflow is intended as a practical summary of our observed rule-in pattern and read-count-dependent certainty, rather than rather than confirmatory. In particular, higher max reads may strengthen confidence in a clinically concordant positive result, but they should not be treated as standardized cross-platform decision thresholds.

## 4 Discussion

This study evaluated CSF mNGS in a consecutive single-center cohort of adults with suspected CNS infection and found that positive results were more informative for supporting infection than negative results were for excluding it. The observed pattern of higher positivity in Definite than Probable or Unlikely cases is clinically plausible and consistent with prior reports that sequencing performs best when there is stronger underlying evidence of infection. At the same time, the modest sensitivity and negative predictive value in our cohort indicate that a negative CSF mNGS result should not provide false reassurance when clinical suspicion remains high.

Among the 46 patients with suspected CNS infection, CSF metagenomic sequencing detected pathogens in 28.3% of samples. Positivity differed markedly across adjudicated categories, occurring in 73.3% of Definite cases, 11.8% of Probable cases, and none of the Unlikely cases. This pattern is consistent with previous studies showing that unbiased sequencing can provide clinically useful diagnostic information, particularly when conventional testing is unrevealing, while still requiring careful interpretation in the clinical context (Ramachandran and Wilson, 2020; Wilson et al., 2019). Using Likely etiology as the composite reference standard, the assay showed 100% specificity and 100% positive predictive value, whereas sensitivity was lower at 40.6%. Taken together, these findings suggest that CSF metagenomic sequencing is most helpful for confirming infection when positive, but a negative result should not be used to exclude disease.

Our findings also suggest that a simple patient-level max-read summary may help contextualize positive results, but only in a limited exploratory sense. Read counts are not directly comparable across organisms, platforms, sample types, or laboratory pipelines, and polymicrobial reports complicate simple quantitative attribution. For these reasons, the proposed <10, 10-49, and >=50 categories should be viewed as pragmatic descriptive bins rather than validated clinical thresholds.

The study also highlights several interpretive challenges. More than half of patients with negative sequencing results were still ultimately considered to have an infectious etiology, indicating a substantial false-negative risk. Several factors may contribute to this. Pathogen burden in CSF may be low, especially when sampling occurs late in the disease course or after partial treatment. In addition, technical factors, including nucleic acid recovery and library preparation, may affect analytical yield. Prior studies have also shown that sequencing performance can vary depending on whether cell-free or cellular nucleic acid workflows are used in neuroinfectious disease testing (Xing et al., 2020).

Antimicrobial exposure before lumbar puncture was common in our cohort and may have reduced pathogen detection. Previous studies in other infectious settings have reported lower sequencing yield after even brief treatment exposure, making it plausible that prior antimicrobial therapy contributed to some of the negative results observed here (Hamilton et al., 2020; Hao et al., 2024; Yu et al., 2021). Accordingly, negative mNGS results should be interpreted together with CSF cell counts, neuroimaging, conventional microbiology, and follow-up assessment rather than in isolation. Repeat sampling may also be reasonable when clinical suspicion remains high (Chen et al., 2022).

The pathogen spectrum identified in this study was also clinically informative. The predominance of HSV-1 and VZV is in line with the recognized epidemiology of viral CNS infection and supports the role of CSF metagenomic sequencing as an adjunctive tool in suspected viral encephalitis. Recent reports likewise highlight the importance of VZV in neurologic infection (Tang et al., 2024). By contrast, EBV detection in CSF requires more cautious interpretation, as it may reflect true neuroinvasive disease, viral reactivation, or incidental shedding depending on the host immune state and overall clinical syndrome (Ramachandran and Wilson, 2020). The identification of Balamuthia mandrillaris further illustrates one of the major advantages of metagenomic approaches: the ability to detect unexpected or difficult-to-culture pathogens that may not be captured by routine diagnostic strategies. At the same time, such findings still require clinical correlation and, where possible, orthogonal confirmation (Ramachandran and Wilson, 2020; Wilson et al., 2019).

Our findings also have potential implications for clinical interpretation at the bedside. Positive CSF metagenomic sequencing results, particularly those with higher read counts, can strengthen an infectious hypothesis, whereas negative results should be interpreted cautiously. We therefore view read-count stratification as a practical interpretive aid rather than a stand-alone diagnostic rule. Because read counts are not directly comparable across organisms and because our positive subgroups were small, these proposed thresholds should be considered preliminary. Further multicenter studies with standardized reference standards, more detailed documentation of antimicrobial exposure, and harmonized quantitative reporting will be needed to determine whether this approach is reproducible and clinically robust.

Several limitations warrant emphasis. First, this was a single-center retrospective study with only 13 positive mNGS cases, limiting precision and making the read-count analyses unstable. Second, the composite clinical reference standard was partly subjective and not fully independent of the index test. Because adjudicators were not blinded to routine-care mNGS findings, incorporation bias may have inflated apparent concordance, specificity, and positive predictive value. Third, not all cases had uniform orthogonal microbiological confirmation. This issue has been addressed differently in some recent studies that excluded metagenomic findings from the reference standard (Zhao et al., 2025). Fourth, frequent antimicrobial treatment before sampling may have lowered the diagnostic yield and contributed to false-negative results (Chen et al., 2022; Hao et al., 2024). Fifth, all samples underwent DNA-based testing only, so the study cannot assess RNA-virus-related CNS infection or represent the performance of combined DNA/RNA mNGS workflows. Finally, selection bias remains possible because mNGS is often preferentially ordered in diagnostically difficult patients. Finally, we did not incorporate relative abundance measures or formal background-control metrics, which may limit the ability to distinguish low-level true signals from contamination on the basis of quantitative data alone.

Despite these limitations, the results support a cautious role for CSF mNGS as an adjunctive diagnostic test in suspected CNS infection. A clinically concordant positive result may strengthen the likelihood of infection, whereas a negative result cannot reliably exclude it. Larger multicenter studies with standardized reference standards, better documentation of pre-sampling therapy, and harmonized quantitative reporting will be required before any read-count-based interpretive scheme can be considered robust.

## 5 Conclusions

In this real-world DNA-based cohort, positive CSF mNGS findings supported an infectious etiology more strongly than negative findings excluded it. Patient-level max-read stratification may offer exploratory interpretive value for positive results, but it should be regarded as preliminary and hypothesis-generating rather than as a validated decision framework. Further multicenter studies are needed to refine these observations using more standardized adjudication procedures and broader molecular testing strategies.

## Data Availability

The data analyzed in this study was obtained from The First Affiliated Hospital of Bengbu Medical University, the following restrictions apply. Requests to access these datasets should be directed to Di Nian, niandi07@sohu.com.

## References

[B1] Chen H. Zheng Y. Zhang X. Liu S. Yin Y. Guo Y. . (2024). Clinical evaluation of cell-free and cellular metagenomic next-generation sequencing of infected body fluids. J. Adv. Res. 55, 119–129. doi: 10.1016/j.jare.2023.02.018 36889461 PMC10770109

[B2] Chen Y.-Y. Guo Y. Xue X.-H. Pang F. (2022). Application of metagenomic next-generation sequencing in the diagnosis of infectious diseases of the central nervous system after empirical treatment. WJCC 10, 7760–7771. doi: 10.12998/wjcc.v10.i22.7760 36158512 PMC9372857

[B3] Hamilton W. L. Pires S.-M. Lippett S. Gudka V. Cross E. L. A. Llewelyn M. J. (2020). The impact of diagnostic microbiology on de-escalation of antimicrobial therapy in hospitalised adults. BMC Infect. Dis. 20, 102. doi: 10.1186/s12879-020-4823-4 32013908 PMC6998081

[B4] Hao L. Bian W. Qing Z. Ma T. Li H. Xu P. . (2024). Will previous antimicrobial therapy reduce the positivity rate of metagenomic next-generation sequencing in periprosthetic joint infections? A clinical study. Front. Cell. Infect. Microbiol. 13, 1295962. doi: 10.3389/fcimb.2023.1295962 38274732 PMC10808557

[B5] Neuhäuser M. Hothorn L. A. (1999). An exact Cochran–Armitage test for trend when dose–response shapes are a priori unknown. Comput. Stat. Data Anal. 30, 403–412. doi: 10.1016/S0167-9473(98)00091-7

[B6] Ramachandran P. S. Wilson M. R. (2020). Metagenomics for neurological infections — expanding our imagination. Nat. Rev. Neurol. 16, 547–556. doi: 10.1038/s41582-020-0374-y 32661342 PMC7356134

[B7] Shean R. C. Garrett E. Malleis J. Lieberman J. A. Bradley B. T. (2024). A retrospective observational study of mNGS test utilization to examine the role of diagnostic stewardship at two academic medical centers. J. Clin. Microbiol. 62, e0060524. doi: 10.1128/jcm.00605-24 39162437 PMC11389146

[B8] Tang J. Wang K. Xu H. Han J. (2024). Metagenomic next-generation sequencing of cerebrospinal fluid: a diagnostic approach for varicella zoster virus-related encephalitis. Front. Cell. Infect. Microbiol. 14, 1509630. doi: 10.3389/fcimb.2024.1509630 39776437 PMC11703743

[B9] Team C (2012). “ Team RDC.R: A language and environment for statistical computing,” in R Foundation for Statistical Computing ( R: A Language and Environment for Statistical Computing, Vienna, Austria). doi: 10.32614/r.manuals

[B10] Volpe B. T. (2022). Editorial: Central nervous system inflammatory injury, and now in the time of epidemic. Curr. Opin. Neurol. 35, 373–374. doi: 10.1097/WCO.0000000000001056 35674082 PMC9197200

[B11] Wang J. Ye J. Yang L. Chen X. Fang H. Liu Z. . (2022). Inconsistency analysis between metagenomic next-generation sequencing results of cerebrospinal fluid and clinical diagnosis with suspected central nervous system infection. BMC Infect. Dis. 22, 764. doi: 10.1186/s12879-022-07729-0 36180859 PMC9523998

[B12] Wilson M. R. Sample H. A. Zorn K. C. Arevalo S. Yu G. Neuhaus J. . (2019). Clinical metagenomic sequencing for diagnosis of meningitis and encephalitis. N. Engl. J. Med. 380, 2327–2340. doi: 10.1056/NEJMoa1803396 31189036 PMC6764751

[B13] Wu J. Song W. Yan H. Luo C. Hu W. Xie L. . (2024). Metagenomic next-generation sequencing in detecting pathogens in pediatric oncology patients with suspected bloodstream infections. Pediatr. Res. 95, 843–851. doi: 10.1038/s41390-023-02776-y 37857845 PMC10899103

[B14] Xing X.-W. Zhang J.-T. Ma Y.-B. He M.-W. Yao G.-E. Wang W. . (2020). Metagenomic next-generation sequencing for diagnosis of infectious encephalitis and meningitis: A large, prospective case series of 213 patients. Front. Cell. Infect. Microbiol. 10, 88. doi: 10.3389/fcimb.2020.00088 32211343 PMC7066979

[B15] Yu J. Diaz J. D. Goldstein S. C. Patel R. D. Varela J. C. Reyenga C. . (2021). Impact of next-generation sequencing cell-free pathogen DNA test on antimicrobial management in adults with hematological Malignancies and transplant recipients with suspected infections. Transplant. Cell. Ther. 27, 500.e1–500.e6. doi: 10.1016/j.jtct.2021.02.025 33849818

[B16] Yu L. Zhang Y. Zhou J. Zhang Y. Qi X. Bai K. . (2022). Metagenomic next-generation sequencing of cell-free and whole-cell DNA in diagnosing central nervous system infections. Front. Cell. Infect. Microbiol. 12. doi: 10.3389/fcimb.2022.951703 36237422 PMC9551220

[B17] Zhao Z. Zhang Y. Fu J. Yu L. (2025). Metagenomic next-generation sequencing for cryptococcal meningitis diagnosis: a single-center experience. Front. Cell. Infect. Microbiol. 15, 1626290. doi: 10.3389/fcimb.2025.1626290 41459152 PMC12738950

